# HIV Testing and Counseling Among Female Sex Workers: A Systematic Literature Review

**DOI:** 10.1007/s10461-018-2043-3

**Published:** 2018-02-20

**Authors:** Anna Tokar, Jacqueline E. W. Broerse, James Blanchard, Maria Roura

**Affiliations:** 1 ISGlobal, Barcelona Institute for Global Health, University of Barcelona, Hospital Clínic - Universitat de Barcelona, Rosselló, 132, 08036 Barcelona, Spain; 2 Athena Institute, Faculty of Science, Vrije Universiteit Amsterdam, Amsterdam, The Netherlands; 3 Department of Community Health Sciences, Manitoba University, Manitoba, Canada; 4 Public and Patient Involvement Research Unit, Graduate Entry Medical School, University of Limerick, Limerick, Ireland

**Keywords:** HIV diagnosis, HIV testing, Female sex workers (FSWs), Systematic review

## Abstract

HIV testing uptake continues to be low among Female Sex Workers (FSWs). We synthesizes evidence on barriers and facilitators to HIV testing among FSW as well as frequencies of testing, willingness to test, and return rates to collect results. We systematically searched the MEDLINE/PubMed, EMBASE, SCOPUS databases for articles published in English between January 2000 and November 2017. Out of 5036 references screened, we retained 36 papers. The two barriers to HIV testing most commonly reported were financial and time costs—including low income, transportation costs, time constraints, and formal/informal payments—as well as the stigma and discrimination ascribed to HIV positive people and sex workers. Social support facilitated testing with consistently higher uptake amongst married FSWs and women who were encouraged to test by peers and managers. The consistent finding that social support facilitated HIV testing calls for its inclusion into current HIV testing strategies addressed at FSW.

## Resumen

La aceptación a realizar las pruebas de VIH continúa siendo baja entre las Mujeres Trabajadoras Sexuales (MTS). Nosotros sintetizamos evidencias sobre las barreras y las facilidades para realizar las pruebas de VIH entre las MTS, así como sobre las frecuencias de la prueba, voluntad de evaluar y tasa de retorno para recoger los resultados. Se realizaron búsquedas sistemáticas en las bases de datos MEDLINE/PubMed, EMBASE,SCOPUS para artículos publicados en inglés entre enero del 2000 y noviembre de 2017. De 5036 referencias examinadas, elegimos 36 artículos. Las dos barreras más comunes para las pruebas de VIH fueron los costos financieros y de tiempo, incluyendo: bajos ingresos, costos de transporte, limitaciones de tiempo y pagos formales/informales, así como el estigma y la discriminación atribuidos a las personas y trabajadoras sexuales seropositivas. El apoyo social facilitó las pruebas de VIH con una mayor aceptación entre las MTS casadas y las mujeres a quienes sus compañeros y gerentes les animaron a realizarlas. El hallazgo consistente en que el apoyo social facilitó las pruebas de VIH requiere su inclusión en las estrategias actuales de pruebas de VIH realizadas en MTS.

## Introduction

Worldwide, early HIV testing is a public health priority especially among key populations such as female sex workers (FSWs) [1–3]: out of the estimated 33 million people living with HIV in the world, 19 million do not know their status [1]. Early HIV diagnosis has gained significant attention within key global health institutions, including the Joint United Nations Program on HIV/AIDS (UNAIDS) and the recently established 90-90-90 targets [4]. It is proposed that by 2020, 90% of all people living with HIV should know their HIV status, 90% of all people with diagnosed HIV should receive sustained antiretroviral treatment, and 90% of all people receiving antiretroviral treatment should reach viral suppression [4]. Historically, HIV prevention efforts focused on key populations, including sex workers, as an effective approach to reduce HIV transmission, particularly in the early phase of the epidemic [5].

Several systematic reviews have examined HIV prevalence [6–8] and effectiveness of different HIV prevention interventions for SWs [9–12]. Shahmanesh et al. presented evidence for the efficacy of multi-component interventions, and⁄or structural interventions [9]. A Cochrane review of behavioral interventions concluded that, compared with standard care or no intervention, behavioral interventions are effective in reducing HIV and the incidence of STIs amongst FSWs [10]. A systematic review of community empowerment interventions in low- and middle-income countries demonstrated significant protective combined effect for HIV infection (prevalence), STIs such as gonorrhea and chlamydia, and increase of consistent condom use with all clients [12]. A systematic review of community empowerment interventions in generalized and concentrated epidemics has shown their positive impact on HIV prevalence, estimated number of averted infections among SWs and adult population, and expanded coverage of ART [11]. These previous studies did not systematically assess HIV testing approaches, but rather examined the combined effect of a variety of prevention activities. Thus, they failed to address unique determinants of different HIV testing approaches.

HIV testing activities among sex workers were assessed in only two papers including a meta-analysis of community-based approaches [13], and a study of barriers to HIV testing in Europe [14]. According to these studies, community-based HIV testing leads to higher HIV testing rates than facility-based testing, and the most common barriers to HIV testing are low-risk perception, fear and worries, poor accessibility to healthcare services, health providers’ reluctance to offer the test, and scarcity of financial and human resources. Still, neither of those studies focused on FSWs nor systematically reviewed unique facilitators and barriers to HIV testing faced by this group. The present review compiles existing evidence on HIV testing among FSWs in order to better meet the needs of this group while implementing the first target of the 90-90-90 strategy. Our specific objectives are: (1) to summarize data on key barriers and facilitators to HIV testing among FSWs, and (2) to systematically review frequencies of testing, willingness to test, and return rates to collect HIV test results in this population.

## Methods

We applied a free text strategy and MeSH terms to systematically scan the electronic databases MEDLINE/PubMed using the platform OVID, and EMBASE and SCOPUS. We employed a combination of terms that covered the concepts ‘HIV’, ‘Sex work’ and ‘Test’. We conducted several scoping searches to identify the most efficient search strategy, which we provide in “Annex 1: Search strategy”. Guidelines, reports and policy documents were searched using Google Scholar and employed to inform the discussion of findings. We exported all identified references (5036) into the bibliographic management software ENDNOTE X7.

**Fig. 1:**
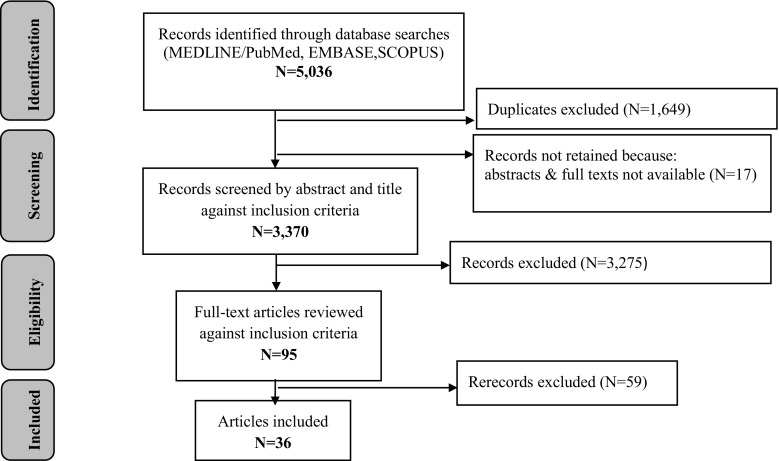
PRISMA flow chart of citations

The first author (AT) screened titles and abstracts against the following inclusion criteria: (1) published in a peer-reviewed journal between January 2000 and November 2017; (2) written in English; and (3) presenting data on HIV testing among FSWs. We excluded duplicates and studies for which no abstract or full text was available (N = 17). After reviewing the full text of 95 pre-selected articles against the above mentioned inclusion criteria, 36 papers were retained for a more detailed review. The first author extracted data systematically using a standardized form that included information on the period of study, location, study population, design, research questions, key findings, and conclusions (“Annex 2: Data extraction form”). Next the quality of qualitative papers was assessed using the guide for critically appraising qualitative research by Spencer et al. [15]. The modified *Downs and Black* checklist was applied to quantitative and mixed-methods papers [16]. We used a midpoint score of 9 for qualitative papers and 12.5 for quantitative ones as a cut off between low- and high-quality studies. Overall, two quantitative papers with score of 10 [18] and 9 [17] points failed to meet the criteria; three papers received 12 points [19, 20]. The vast majority of quantitative papers lacked information needed for assessment. We were unable to appraise four abstracts: three, for the limited data presented, and one, being a mathematical modeling paper [21] that did not fit well with the quality appraisal tools employed. We decided to include all papers into the review in order to provide a comprehensive picture; at the same time, we considered it important to stress the results of the quality assessment (“Annex 3: Quality assessment”). The results of the search and screening process are described in Fig. 1.

Guided by the socio-ecological model (SEM) developed by Blanchard et al. [22] we classified data into three levels: macro-, meso-, and micro-level factors (Table 1). The macro level consisted of economic and policy factors. The meso level included social networks, organizations, cultural norms, and values. The micro level included individual socio-demographic characteristics, knowledge, risk awareness, and behavioral factors. We also extracted data on previous experiences of HIV testing and ways to encourage uptake. The PRISMA check list is provided as “Annex 4: PRISMA 2009 Checklist”.

## Results

Out of the 36 studies retained for review, most were quantitative (N = 20) and conducted in Asia (N = 18). Nine papers reported work conducted in Africa, three in Europe, three in Russia, and one in Macedonia. Three studies were conducted in Latin America, two in Canada and one in Australia. Eighteen studies were cross-sectional, and twenty-five focused exclusively on FSWs.

### Previous Experience of HIV Testing

We summarized evidence on previous experience of HIV testing and approaches to facilitate testing (Table 1). Fifteen studies [17, 18, 23–35] focused on ever in life testing. The highest rate was reported in a study in Russia where all recruited FSWs (100%, N = 29) were tested [24] followed by Kenya (88.6%, N = 818) [27]. The lowest rate was reported in a study conducted in India (7.9%, N = 6648) [18]. Ten studies examined recent testing [25, 27–29, 35–40], which varied extensively from 76.1% in Canada (N = 435) [25] to 22% in China (N = 970) [38]. Six studies addressed willingness to test [18–20, 28, 41, 42], and this ranged from 88% (N = 17) in China [41] to 73.2% in India (N = 6648) [18]. Three studies reported that willingness facilitated actual HIV testing [20, 28, 41]. Only four studies assessed frequencies of collecting test results [23, 26, 33, 43], and these ranged widely from 92.3% in Guinea [26] to 14.8% in Thailand [43].

**Table 1:** Overview of selected studies

Study reference	Location	Study population (N, population group)	Study design	HIV testing uptake	Micro-level factors	
					Barriers	Facilitators
1. Aho et al. [26]	Guinea	N = 421 FSWs	Mixed (QN,CS & QL)	Ever tested:26.6% Acceptance:100% Collected result:92.3%	n/a	Higher perceived risk
2. Ameyan et al. [47]	Ethiopia	N = 20 FSWs N = 3 community counsellors N = 3 HCWs N = 3 program managers N = 2 hotel owners	QL	n/a	Lower perceived risk	Young age HIV knowledge Pregnancy, children
3. Batona et al. [28]	Benin	N = 450 FSWs	QN,CS	Ever tested:87% Past year::65.3% Past 3 months:40% 3–6 months:21% Acceptance:98.9% Willing:69.4%	n/a	Previous testing Having kids
4. Beattie et al. [48]	India	N = 302 FSWs = 125 MSM = 56 TSG = 6 Female peer educators = 87 Male peer educators = 28	QL	n/a	No ill health symptoms Poor VCT knowledge	Improved quality &duration of life (ART) Having kids
5. Bengtson et al. [27]	Kenya	N = 818 FSWs	QN, CS	Ever tested:88.6% Never tested:11% Every 3 months:45% Every 6 months:15% Every 1–3 months:12% Once:16%	31 years and older Less than 7 years of SW Alcohol use	Having kids
6. Burke et al. [54]	Uganda	N = 88 N = 11 FSWs N = 10 Fish men/boat owners N = 12 HCWs N = 55 community members	QL	n/a	n/a	n/a
7. Chanda et al. [49]	Zambia	N = 40 FSWs	QL	n/a	n/a	Pregnancy Protecting one’s child Seeking birth control Family planning Higher HIV knowledge
8. Chiao et al. [45]	Philippines	N = 980 FSWs (baseline) N = 903 FSWs (post test)	QN, randomized quasi-experimental	n/a	n/a	Older age Better educated Longer employed in SW Regular SP Higher income Higher HIV knowledge Higher perceived risk
9. Dandona et al. [18]	India	N = 6648 FSWs	QN,CS	Ever tested:7.9% Unwilling:73%	16–17 years old Low income	Engaged in SW > 5 years Higher income
10. Deering et al. [37]	Canada	N = 291 SWs	QN,CS	Past year: 69.4%	n/a	n/a
11. Deering et al. [25]	Canada	N = 435 SWs	QN,CS	Ever tested:87.4% Past year:76.1%	Migrant/new migrant Language barriers	Older age of SW initiation Aboriginal ancestry Inconsistent condom use with clients Injecting drugs Contact with nursing program
12. Dugas et al. [29]	Benin	N = 66 FSWs N = 24 HCWs	QL	Every 6 months:46% Never tesed:26%	No symptoms Migration Language barriers	n/a
13. Grayman et al. [30]	Vietnam	N = 610 FSWs	QN,CS	Ever tested:30.9%	Never married 5 or fewer clients per week	n/a
14. Hong et al. [31]	China	*N* = 1022 FSWs	QN,CS	Ever tested:48%	Lower perceived risk Inconsistent condom use with client/stable SP Poor VCT knowledge	Higher HIV knowledge Older age Low education
15. King et al. [46]	Russia	N = 29 FSWs	QL	Ever tested:100%	Drug use Poverty	Having money
16. King et al. [46]	Russia	N = 139 FSWs	QN,CS	n/a	Older age Longer duration of drug use	n/a
17. King et al. [34]	Russia	N = 139 FSWs N = 29 FSWs	Mixed (QN,CS& QL)	Ever tested: 97–100%	Drug use Drug use for ≥ 4 years Poor knowledge where to test	Higher HIV knowledge Perceived risk High HIV incidence Knowing someone who is HIV+ Wanting to protect others (children, parents)
18. Johnston et al. [40]	Dominican Republic	N = 2781 FSWs	QN, CS	Past year: 16.6–30.8%	n/a	Higher HIV knowledge Regular HC checks-
19. Ngo et al. [50]	Vietnam	N = 30 FSWs (in-depth interview) N = 94 FSWs (FGDs)	QL	n/a	Poor VCT knowledge Perceived risk	Fear of HIV Visual STI symptoms Drug use of intimate SP
20. Nhurod et al. [43]	Thailand	N = 1006 FSWs	QN,CS	Acceptance:91.2% collected result:at clinic-100% at mobile point-14.8%	n/a	n/a
21. Park et al. [38]	China	N = 348 FSWs	QN,CS	Past year:22%	n/a	Higher HIV knowledge Participation in HIV prevention program Income Education Regular STI checks
22. Parriault et al. [17]	Boarder between Brazil and French Guiana	N = 213 FSWs	QN, CS	Never tested: 31%	Older age HIV knowledge Poor knowledge where to test Lower perceived risk	n/a
23. Sayarifard et al. [36]	Iran	N = 128 FSWs	QN,CS	Past year:25%	Poor knowledge	n/a
24. Scorgie et al. [52]	four countries of east and southern Africa (Kenya, Zimbabwe, Uganda and South Africa)	N = 106 FSWs N = 26 MSWs *N* = 4 TSG	QL	n/a	Poor VCT knowledge	n/a
25. Shokoohi et al. [35]	Iran	N = 1005 FSWs	QN, CS	Past year 27.5%	n/a	Older age at first SW HIV knowledge Knowledge where to test Injecting drugs ever in life Receiving free condoms during 12 months Higher perceived risk
26. Shokoohi et al. [39]	Iran	N = 1337 FSWs	QN, CS	Ever tested:80.6% Past year 70.4%	n/a	Higher educational level Higher HIV knowledge Knowledge of where to test More than 5 paying SP More than one none-paying SP Consistent condom use Receiving HR services Health service utilization Higher perceived risk
27. Simonovikj et al. [53]	Macedonia	N = 106 SWs	Case report	n/a	n/a	n/a
28. Todd et al. [32]	Uzbekistan	N = 448 FSWs	QN,CS	Ever tested:83.9%	Younger age Initiated SW before age 18 Shared drugs with clients Consistent condom use with clients Shorter period of SW Fewer SP Poor VCT knowledge	n/a
29. Tran et al. [33]	Vietnam	N = 1998 FSWs	QN,CS	Ever tested: 34.4% ESWs 24.4%-SSWs Collected result:86.9%	Ever injected drugs Having IDUs as clients Inconsistent condom use with non-commercial SP lover and husband)	Duration of SW Inconsistent condom use with clients Higher HIV knowledge (information via communication campaign) Higher perceived risk
30. Wang et al. [41]	China	N = 17 FSWs N = 12 pimps	QL	Willing:88%	Poor HIV knowledge (receiving information through TV on decrease of HIV P) HIV negative-peers/friends Lower perceived risk Consistent condom use	Higher risk awareness (receiving information through TV on increase of HIV P) Someone close diagnosed HIV+ Concerned about friends and family Concerned about one’s health
31. Wang et al. [19]	China	N = 970 FSWs	QN,CS	Willing:69% Unwilling:7.2%	n/a	Married Engaged in SW for longer Higher VCT knowledge Higher perceived risk Condom use Leave SW Previous HIV testing
32. Wang et al. [20]	China	N = 970 FSWs	QN, Prospective cohort	Willing:69% Acceptance:11%	Lower perceived risk	Engaged in SW > 12 months Condom use Willing to test Changing job
33. Wanyenze et al. [51]	Uganda	*N* = *190* FSWs	QL	n/a	Poor HIV knowledge Misconceptions Limited awareness of services	n/a
34. Wilson et al. [21]	Australia	N = n/a SWs	Mathematical model	n/a	n/a	n/a
35. Xun et al. [42]	China	N = 371 MSM N = 405 FSWs *N* = 361 VCT clients	QN,CS	Willing:72.1% -54.3% at home	n/a	Willing to pay 4.8 USD for oral test Higher risk awareness
36. Xu et al. [23]	China	N = 164 FSWs	QN longitudinal	Ever tested:32.1%-HIV+ , 16.1%-HIV− Collected result:47.8%	n/a	More than 9 years of schooling Less than 5 clients per week Having a regular SP Drug use Pelvic pain during last 12 months Higher perceived risk

Study reference	Meso-level factors		Macro-level factors		Strategies to encourage HIV testing
	Barriers	Facilitators	Barriers	Facilitators	
1. Aho et al. [26]	HIV stigma SW stigma Managers’ negative attitude	Peer support	n/a	Free treatment	n/a
2. Ameyan et al. [47]	HIV stigma and discrimination Time restriction Higher travel costs Negative attitude of HCWs Negative experience of peers/brothel owners	Private hospitals Financial incentives	Need to show ID Poor confidentiality	Free treatment and other HC services	Involvement of brothel owners Involvement of media Media
3. Batona et al. [28]	n/a	n/a	n/a	n/a	n/a
4. Beattie et al. [48]	Fear of HIV+ result, HIV +status/SW disclosure Fear to leave the brothel (violence) Discrimination of FSWs/HIV+ Higher travel costs Time costs	Peer support	Lack of confidentiality Bribes ID and retention Need to have retention “buddy”	n/a	Peer educators
5. Bengtson et al. [27]	n/a	n/a	n/a	n/a	n/a
6. Burke et al. [54]	n/a	n/a	n/a	n/a	Self-testing
7. Chanda et al. [49]	Fear of stigma and discrimination HIV stigma SW stigma Intimate partner violence Potential financial Time restrictions	Community/close social network	Fear of poor confidentiality	n/a	Community empowerment Norm-changing interventions
8. Chiao et al. [45]	n/a	Support from peers and managers	n/a	n/a	Education of peers and managers
9. Dandona et al. [18]	Non-participation in self support group	Non street-based Participation in support group	n/a	n/a	n/a
10. Deering et al. [37]	n/a	High density of testing sites Lower travel costs	n/a	n/a	n/a
11. Deering et al. [25]	Indoor SW	n/a	Criminalization of SW	n/a	n/a
12. Dugas et al. [29]	Fear of HIV+ test result Fear of SW disclosure Low quality of HC	Peer-educators Pimps’ support	n/a	n/a	Peer educators Home/work testing Community mobilization
13. Grayman et al. [30]	n/a	n/a	n/a	Time ever spent in rehabilitation centre for drug addicts	n/a
14. Hong et al. [31]	Fear of HIV+ status disclosure Fear of SW disclosure Higher time costs	Working at high income venues	n/a	n/a	n/a
15. King et al. [46]	Perceived low quality of public HC services Social marginalization	Family support Having relatives employed as medical professionals Personal “connections” with HC system Referrals of NGOs More accessible private hospital	Bribes Residence permit Fear of registration as drug user, STI patient or HIV+ in official medical records Need to give up possibility of anonymity Underfunding of HC Illegal SW	Outreach program/referrals	Outreach with the referrals
16. King et al. [46]	HIV stigma Refusal of medical care (IDUs, FSWs)	SW stigma	Poor confidentiality	n/a	n/a
17. King et al. [34]	Higher time and travel costs Fear of HIV+ test result SP reaction if tested HIV+ Stigma and discrimination	Social support Perceived better conditions in private clinics	Poor confidentiality High costs residence permit Fear of registration as drug user, STI patient or HIV+ in official medical records	Free treatment Outreach van	Outreach testing
18. Johnston et al. [40]	n/a	Social support	n/a	n/a	n/a
19. Ngo et al. [50]	Street based SW Higher time costs (waiting lists) Lack of money&time Fear of HIV+ result Fear to be recognized as SW Discrimination, unfriendly perceived low quality of public hospitals Fear of imprisonment if diagnosed HIV+	Venue-based SW Managers requirement to test Perceived high quality (no waiting, less of private) private HC facilities	Poor confidentiality, HC costs at private hospitals	Less perceived discrimination at private clinics Free treatment Abortion, giving birth or rehabilitation centre for drug addicts	Involvement of managers Peer-referrals
20. Nhurod et al. [43]	Higher time costs Money needed to travel to collect test result check	Required negative result to be employed as SW	n/a	n/a	Mobile VCT Rapid testing
21. Park et al. [38]	n/a	SW venue	n/a	n/a	Cooperation of NGO and FSWs’ network
22. Parriault et al. [17]	n/a	n/a	n/a	n/a	n/a
23. Sayarifard et al. [36]	Fear of HIV+ result Fear of result disclosure	n/a	Higher costs	n/a	Training program for FSWs
24. Scorgie et al. [52]	Self-stigma Fear of HIV+ result Fear of isolation and discrimination Higher transportation costs	NGO established clinic Perceived higher quality of private HC facilities	Forced testing without consent Denial of testing Illegal SW	n/a	Training of health providers Supporter or advocate in HC facilities Collective action of SWs
25. Shokoohi et al. [35]	n/a		n/a	n/a	Rapid testing
26. Shokoohi et al. [39]	n/a	n/a	n/a	n/a	Referral’s Community-based outreach testing Self-testing
27. Simonovikj et al. [53]	n/a	n/a	Cases of forced testing during police arrest	n/a	n/a
28. Todd et al. [32]	n/a	n/a	Compulsory testing during police detainment Illegal SW Fees for testing and treatment	n/a	Change in criminal law Guarantee anonymous testing Establishment of “friendly cabinets”
29. Tran et al. [33]	n/a	n/a	54% were tested voluntarily, other were forced	n/a	n/a
30. Wang et al. [41]	Fear of HIV+ result Meeting friends at VCT sites Fear of HIV+ diagnosis Lack of confidentiality Fear of HIV + disclosure Fear of sex work disclosure Stigma Higher time costs	Support from peers and managers	Fear of being quarantined Higher medical cost Mistrust in governmental free services	n/a	Education information Dissemination
31. Wang et al. [19]	Stigmatization and discrimination	Support from peers, family, and managers	n/a	Free treatment	Peer education -VCT promotion
32. Wang et al. [20]	Stigmatization and discrimination SW disclosure	Support from peers	n/a	Free treatment	Peer education
33. Wanyenze et al. [51]	Fear of stigma and discrimination Unwelcoming attitude of HC workers and discrimination Stigmatization by fellow sex workers, families and the community Unfavourable opening hours of health facility The pace at which HIV services are delivered	Social networks	Costs (“tips” to healthcare providers) Fear of breach of confidentiality Illegal SW	Faster service delivery at private facilities	Use of media Establishing specific facilities or clinics for SWs Provision of health information on phones Motivation and sensitization of HC providers to work with FSWs
34. Wilson et al. [21]	n/a	n/a	Mandatory HIV testing not cost effective	n/a	n/a
35. Xun et al. [42]	n/a	n/a	n/a	n/a	Oral fluid test Home testing
36. Xu et al. [23]	n/a	n/a	n/a	n/a	n/a

*ART* antiretroviral therapy, *CS* cross sectional, *EES* entertainment-based sex workers, *FGDs* focus group discussions, *FSWs* female sex workers, *HC* health care, *HCWs* healthcare workers, *HIV* human immunodeficiency virus, *HIV P* HIV prevalence, *HR* harm reduction, *IDUs* injection drug users, *ID* identity card, *MSM* men who have sex with men, *MSWs* male sex workers, *NGO* non-governmental organizations, *SP* sexual partner, *STI* sexually transmitted infections, *SSWs* street-based sex workers, *SW* sex work, *TSG* transgender, *QN* quantitative, *QL* qualitative, *n/a* non applicable, *VCT* voluntary counselling and testing

We identified a high variability of outcome measures employed in the studies reviewed. For example, the frequency of HIV testing was measured using different time frames and included “last month” and “recent testing” with a time period corresponding to “recent” that varied from 1 year to 1 month.

### Conceptual Framework: Barriers and Facilitators of HIV Testing Amongst FSWs

**Fig. 2:**
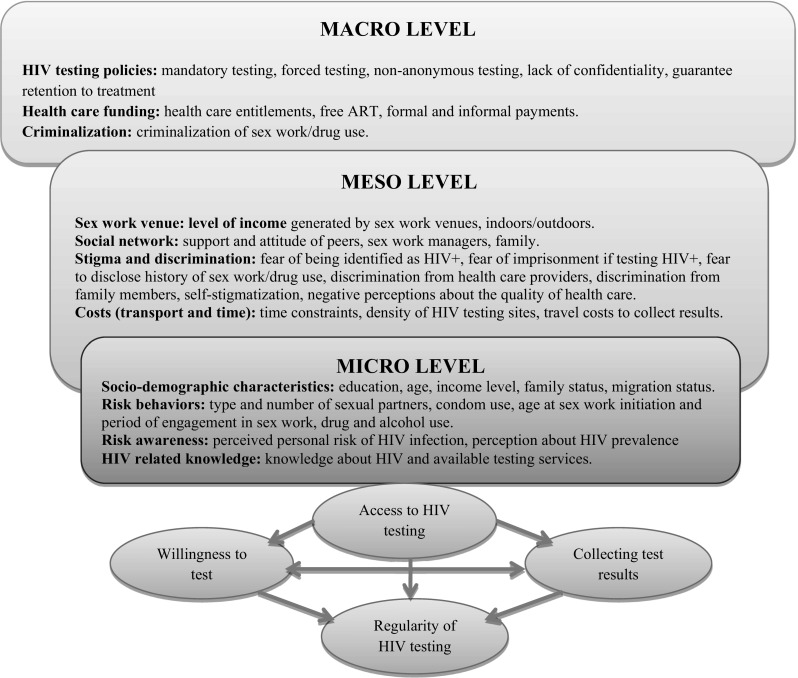
Conceptual framework: barriers and facilitators of HIV testing amongst FSWs. Reproduced with Permission from Blanchard et al. [22]

In this study we employed an adapted version of the socio-ecological framework developed by Blanchard et al. [22] to organize and analyze our findings systematically. As shown in Fig. 2, we conceptualized HIV test uptake as the result of a number of interrelated factors that operate simultaneously at the micro, meso and macro levels. Most articles focused on the micro (N = 30) and meso (N = 24) levels, and about half of all papers addressed the macro (N = 19) level. Ten studies analyzed concurrently two levels of the *social ecology,* while nine addressed simultaneously the micro, meso, and macro levels.

### Micro-level Factors

#### Socio Demographic Characteristics

Sixteen articles focused on socio-demographic characteristics. We found no consistent associations [18, 27, 31, 32, 44–46] of education and age [23, 31, 45] with HIV testing uptake. Highly educated women in the Philippines and Iran were more likely to test [39, 45], but studies in China reported higher HIV testing uptake amongst women with both high [23] and low education level [31]. In two studies conducted in China and one in the Philippines, older age facilitated HIV testing [31, 44, 45], but in Russia, Ethiopia and Kenya older-aged FSWs [46, 47] and those aged + 30 [27] were less likely to test compared to younger FSWs. In Uzbekistan and India, younger age decreased testing [18, 32]. Higher income was associated with testing in India and the Philippines [18, 45] and in Russia poverty impeded access to healthcare, including HIV testing [24].

Ten studies reported that having children and/or being pregnant and/or being in a permanent relationship facilitated HIV testing [19, 23, 27, 28, 30, 34, 45, 47–49]. Married women in China [19] and those with a regular sexual partner in the Philippines [45] and in China [23] were more likely to test. According to a study conducted in Vietnam, unmarried women were less likely to test [30]. In Kenya, Zambia, Benin, Ethiopia, Russia and India, having children or being pregnant facilitated HIV testing [27, 28, 34, 47–49]. In Iran, incarceration was associated with recent testing [39].

In Canada and Benin, migrant FSWs [25, 29] had limited access to healthcare because of language barriers, which led to low HIV testing rates.

#### Risk Behaviors

Seven studies reported nearly inconsistent patterns on how regular condom use influenced HIV testing. Using condoms during every instance of each sexual intercourse both facilitated and impeded testing depending on the type of sexual partner [19, 20, 25, 31, 33, 39, 41]. Three articles reported that inconsistent condom use with a client -facilitated testing [25, 31, 33], but using condoms inconsistently with a husband or lover was negatively associated with testing [33]. In China, a quantitative study identified that condom use was associated with HIV testing [20], although in a previous qualitative study the same author found that FSWs who used condoms consistently felt to be sufficiently protected from HIV and not in need of testing [41]. In Iran, consistent condom use during each sexual intercourse was associated with recent testing [39].

Initiation of sex work at an older age [18, 20, 33, 35, 45] and engaging in sex work for a longer period of time [18, 20, 33, 45] facilitated HIV testing. In Uzbekistan, FSWs who started sex work before the age of 18 were less likely to test [32], and in Canada older age of sex work initiation was positively associated with recent testing [25]. FSWs who engaged in sex work for a longer time had a higher uptake of testing [18, 20, 33, 45] and willingness to test [19]. FSWs employed for shorter periods [27, 32] were less likely to test.

Having a lower number of clients/sexual partners was associated with HIV testing in China [23], but was reported to decrease testing in Vietnam [30], Iran [39] and Uzbekistan [32].

Most studies identified drug use and alcohol consumption to impede HIV testing [24, 25, 27, 32–34, 46]. Still, in Iran and Canada drug use among FSWs did not hinder testing [25, 35].

#### Risk Awareness

We reviewed thirteen articles that focused on individual perceptions towards HIV risks [19, 20, 23, 26, 31, 33–35, 39, 41, 45, 47, 50]. Low perceived risk was associated with lower likelihood to test [31, 45, 47], and FSWs would be less likely to test if they believed that HIV prevalence to be decreasing and no one in their social network was infected [41]. Conversely, high perceived risk was associated with HIV testing [19, 23, 26, 33–35, 39, 41, 45, 50].

#### HIV-Related Knowledge

Seventeen articles examined HIV knowledge, including knowledge of available HIV testing sites in the area [19, 29, 31, 33–36, 38–41, 45, 47–51]. FSWs who had heard prevention messages in HIV communication campaigns were more likely to test [33, 47, 51]. Similarly, FSWs were reluctant to test if they had poor HIV-related knowledge [29, 36, 41, 48, 49, 51] and were not well informed about local testing sites [31, 41, 48, 50–52].

### Meso-level Factors

#### Sex Work Venue

Of the six articles that addressed sex work venues [18, 25, 31, 41, 45, 50], most reported that working indoors and at high-income venues generating higher income impelled HIV testing. Working in a high-income venue and out of the street predicted testing in China, Vietnam and India [18, 31, 50]. However, in Canada, FSWs working indoors were less likely to have recently tested for HIV than those working outdoors [25].

#### Social Support

About half of the articles reviewed assessed how FSWs’ social interactions influenced their decision to test for HIV [18–20, 24, 26, 29, 34, 38, 40, 41, 43, 45, 47–51]. Positive attitudes and support from peers, family and partners facilitated HIV testing [19, 20, 24, 26, 29, 34, 40, 41, 45, 48–51]. In China, women were more likely to test if accompanied by peers [19, 20]. In Russia, family support was an important condition for accessing healthcare, including HIV testing, as women could rely on their family financially and emotionally [24]. Participation in self-support groups in India [18] and Uganda [51] and receiving condoms from HIV prevention programs in China [38] facilitated testing. Positive views of FSWs’ employers towards HIV testing [41, 45] or requiring the test [43, 50] increased the uptake. However, in China and Ethiopia, employers expressed concerns towards HIV testing and how it could impact the sex work business [41, 47]. In Guinea, HIV testing was forbidden by some managers [26]. In Zambia and Russia, fear of negative reaction of their sexual partner if diagnosed HIV-positive, prevented women from engaging in HIV testing [34, 49].

#### Stigma and Discrimination

A total of fourteen articles focused on stigma and discrimination of HIV+ people and/or sex workers [20, 24, 26, 29, 31, 34, 36, 41, 46–52]. Fears of being identified as HIV+ [24, 26, 31, 34, 36, 41, 47–51] or as a sex worker [20, 26, 31, 34, 41, 46–52] were reported as barriers to testing in a number of studies. FSWs could refuse HIV testing [29, 36, 41, 48, 50, 52] or fail to collect test results [48] if afraid of receiving an HIV+ diagnosis.

In Benin, healthcare workers reported that women did not like to be recognized as FSWs, and that this could prevent them from seeking healthcare [29]. In several African countries, health providers discriminated against sex workers and their family members [52]. In Russia, FSWs were concerned that they would be treated badly or denied healthcare if identified as sex workers or drug users [46]. In China, FSWs worried about meeting an acquaintance at the testing site and being recognized as HIV+ [41], while in Vietnam, they feared imprisonment if diagnosed with HIV [50]. Self-stigma resulting from widespread negative views of HIV+ people and sex work decreased testing across several African countries [52]. Thirteen articles reported that anticipated stigma and discrimination at health facilities hampered service utilization [19, 20, 24, 26, 29, 37, 41, 46, 48–52]. In Russia, Vietnam, Uganda and several African countries, private hospitals were defined by FSWs as more friendly and of higher quality than public health facilities, and were reported to be preferable places to get healthcare services, including HIV testing [24, 50–52].

#### Time and Transport Costs

Eleven articles reported that time and transport costs hampered access to healthcare [24, 31, 34, 36, 37, 41, 43, 47, 49–52]. Time restraints impeded testing in China [31, 41], India [48], Thailand [43], Vietnam [50], Uganda [51], Zambia [49], Ethiopia [47] and several other African countries (Kenya, Zimbabwe, Uganda and South Africa) [52]. In Uganda, Zambia and Russia, FSWs expressed dissatisfaction with opening hours of health facilities as they did not correspond with women’s schedules and caused financial and time loss [34, 49, 51]. In Canada, higher density of testing sites and little time needed to get there were associated with having tested recently [37]. In Thailand, travel costs were reported to prevent users from collecting their HIV test results [43].

### Macro Level Factors

#### HIV Testing Policies

Eleven articles focused on HIV testing policies [21, 24, 32, 34, 41, 46–48, 50–52] pointed to shortcomings in the range of services that should be provided together with HIV testing. These include informed Consent, Confidentiality, Counseling, Correct test results, and Connection to care and treatment, known as the “5 Cs” principles [3].

In China [41], Vietnam [50], India [48], Uganda [51], Zambia [49], Ethiopia [47] and Russia [46], lack of confidentiality was reported as a major barrier to HIV testing. Unwillingness to be included in official registers of HIV+ people decreased access to testing in China [41] and Russia [24]. In Russia, FSWs without a residence permit or passport are not entitled to accessing healthcare. Free-of-charge HIV testing is available only upon giving up anonymity, and if a woman utilizes state-sponsored HIV testing at a local clinic, the results are officially recorded into her personal medical records [24]. In India and Ethiopia, to access HIV and AIDS treatment, women are required to show an identity card [47, 48]. In Uganda, all women diagnosed HIV positive were given two papers indicating test result and further referrals while all diagnosed HIV negative were given one paper with test result only [51].

FSWs were forced to test against their will or were tested surreptitiously without consent in Kampala (Uganda), Hillbrow and Limpopo (South Africa) [52] and during police detainment in Uzbekistan [32] and Macedonia [53]. In Vietnam, among the FSWs who tested for HIV, only 54% did it voluntarily [33]. FSWs who had spent time in rehabilitation or detention centers [24, 30, 50] or had ever been pregnant [24, 50] were more likely to have undergone HIV testing. In Victoria, Australia, screening of sex workers is mandatory despite its lack of cost-effectiveness [21].

#### Healthcare Funding

Eleven articles reported how limited healthcare funding decreased HIV testing [19, 20, 24, 26, 32, 36, 41, 48, 50–52]. High medical care costs in China [41], Iran [36], several African countries (Kenya, Zimbabwe, Uganda and South Africa) [52] and informal payments in Russia [24], Uganda [51] and India [48] reduced access to healthcare, including testing. Five articles suggested that free treatment might increase testing [19, 20, 26, 47, 50].

#### Criminalization

Eight studies reported how current criminalized approaches to sex work and drug use inhibited FSWs from accessing healthcare [24, 25, 27, 32, 34, 51, 52, 54]. Sex work criminalization in Kenya, Zimbabwe, Uganda and South Africa, Canada, Uzbekistan and Russia [24, 25, 27, 32, 51, 52, 54] and fear of registration as a drug-user in Russia [24, 34] are seen to have hampered access to healthcare, including testing.

### Testing Modalities

A total of twenty studies examined different ways to encourage HIV testing among FSWs including self-testing [39, 54], home-based [25, 29, 42], rapid testing [35, 43], work-based [29], oral fluid tests [42], “friendly cabinets” (newly established anonymous testing centers) at public STI clinics [32, 51], mobile services [43], outreach with referrals [24, 34, 39], community mobilization [29, 52], community empowerment [49] and involvement of peers, managers and healthcare workers [19, 20, 39, 45, 47, 49–52]. In one study examining hypothetical circumstances around self-testing, participants reported to anticipate significant benefits (entire privacy, avoiding travel and time costs, ability to test before sex early diagnosis), although these same features raised concerns when associated with lack of supportive counseling and linkage to care [54].

These studies suggest that HIV testing might increase if FSWs can easily access testing sites and receive support from peers, friends and healthcare workers along with educational activities [19, 20, 45, 50–52].

## Discussion

This systematic review of barriers and facilitators to HIV testing amongst FSWs found that the two barriers to HIV testing most commonly reported are (1) costs, including transportation, formal/informal payments, and time, and (2) stigma, including fear of involuntary disclosure of HIV status/history of sex work, negative attitudes of healthcare workers, and discriminatory policies. Social support facilitated HIV testing, with consistently higher uptake amongst married FSWs, and those encouraged to test by peers, healthcare workers or employers.

The majority of the studies reviewed were conducted in low and middle-income countries with only three studies identified in high-income settings. Only one study evaluated the cost-effectiveness of HIV testing amongst FSWs. Most studies addressed micro, or micro and meso levels of the SEM with predominance of micro-level factors. Thirteen studies analyzed concurrently the macro, meso and micro levels. Our findings support previous calls to develop HIV testing strategies that fully account for structural factors [55, 56] and highlight the need for a more nuanced investigation of how micro-, meso- and macro-level factors intersect to influence HIV testing uptake.

Few studies assessed frequencies of collecting test results or compared them with testing frequencies. The outcome most frequently assessed was “ever in life” testing although this outcome measure fails to capture the frequency of HIV testing. Furthermore, the highest percentage of recently tested FSWs was reported by a Canadian study and constituted 76.1%, an outcome too low to meet either WHO recommendations [3, 57] or the “90-90-90” target [1], which highlights necessity to increase efforts to promote HIV testing amongst FSWs. Taken together, our results suggest addressing simultaneously several outcome measures when assessing HIV testing programs among female sex workers, including accessibility of testing, willingness to be tested, regularity and collecting test results.

In line with previous studies of HIV testing behavior of different populations [14, 58, 59], we found that scarcity of financial resources, low perceived risk and poor HIV knowledge were barriers to HIV testing for FSWs.

Similar to the results reported for female migrants [58], we observed an association between having children and HIV testing uptake. This might be manifestation of women’s and particularly pregnant women’s greater exposure to HIV testing, as an offer of HIV testing became generally the norm in reproductive and antenatal care settings [3, 60]. On the other hand, several papers reviewed suggested that women in permanent relationships and with children might have higher motivation to stay healthy and thus, might seek out testing themselves. For example, in Vietnam, FSWs in permanent relationships were more likely to be tested in the year 2000 before HIV testing became widely implemented as a part of national antenatal health care program across the country [61]. Overall, social support from family, peers, sex work managers and healthcare workers are instrumental for promoting HIV testing uptake among FSWs, yet the same sources might contribute to further stigma and discrimination.

We did not find any consistent associations between age of participants [58] or their educational level [58, 59] and HIV testing, but working in the sex industry for a longer period and starting sex work at older ages were associated with higher HIV testing uptake. These findings suggest that the willingness to test for HIV might increase with time and relate closely with HIV risk awareness.

The inconsistency of results on how condom use and number of clients influenced testing might be explained by FSWs’ engagement in different types of concurrent sexual partnerships. While using condoms with commercial clients might be perceived as prevailing acceptable behavior [62], the decision to use a condom in cohabiting relationships or with a husband might be influenced by interpersonal factors related to partnership intimacy (e.g., trust, emotional closeness, power or reproductive desires) [63]. Moreover, there is a need to account not only for the type of partnerships, but also for their duration. Consistency of condom use might decrease with longer duration of relationships with non-paying partners [63], but may increase with commercial permanent partners [64]. The relationship intimacy may be at play in the HIV testing decision-making process among FSWs and for consistent condom use. Testing behavior might be influenced by increased trust, emotional closeness and familiarity. A more nuanced understanding of how HIV testing behavior is influenced by risky sexual behaviors in different types of partnerships and how it changes over time is needed. In turn, relationship power might be an important modifiable factor, which might be considered when developing HIV testing interventions for FSWs.

The inconsistencies between results in relation to sexual/drug use behavior and HIV testing might be due to differences in targets of HIV testing approaches across countries. For example, in Canada efforts were concentrated on reaching street-based sex workers and those injecting drugs, leaving out those working indoors, in more high-income venues. Nevertheless, at that time sex work was fully decriminalized in Canada [65]. In contrast, in Uzbekistan and Russia HIV testing might be less accessible for sex workers and drug users because of punitive laws. In these countries, HIV testing is provided solely through government-affiliated settings, including so-called “friendly cabinets” and thus, sex workers and drug users might avoid state clinics or at least avoid disclosing who they are, as they might be stigmatized by healthcare providers or even arrested. Our results demonstrate how laws might diminish promising health-promoting interventions in some countries while in others, supporting policies and concentrated efforts might lead to the successful enrolment of most vulnerable populations.

Furthermore, factors, such as violations of human rights when forcing FSWs to test, lack of confidentiality and anonymity, discriminatory attitudes of healthcare workers, fear of testing HIV+ and being identified as a sex worker and/or a drug user, are manifestations of prevailing stigma. Unfortunately, there are still cases where the violation of basic human rights is “justified” and sex workers are perceived as victims and objects of pity, who should be helped when applying mandatory or forced testing. Our findings highlight the importance to tackle overlapping stigma and discrimination across all three levels of SEM in order to promote HIV testing among sex workers [66]. This is in line with the WHO’s call to enforce the 5 Cs principles and to institutionalize policies preventing discrimination and promoting tolerance towards sex workers and people living with HIV [61, 67]. As reported before, introduction of discriminative laws and policies criminalizing sex work and/or HIV transmission may fuel stigma [65, 68–70].

This review has several limitations. It is restricted to studies published in English, but only three pre-selected studies were excluded for this reason, so the impact upon the findings is likely to be minimal. We included studies published during the last 17 years to account for recent HIV testing approaches. It is unlikely that the content of previously published articles would have substantially altered our findings, as rapid HIV testing started in the early 2000s. We excluded eight citations with neither abstract, nor title available. We acknowledge that our findings are based on the topics presented by the selected studies, and thus, are restricted by the reported information. Despite the limitations mentioned above, this study provides a broad overview of the different aspects of HIV testing across the global SEM, provides important insights on how HIV testing uptake could be promoted among FSWs, and suggests avenues for further research.

## Conclusion

The consistent finding that social support facilitated HIV testing calls for the inclusion of meso- level factors into current HIV testing strategies directed at FSW. Studies on the role of macro-level factors and their intersections with the meso and micro levels are needed to inform interventions that facilitate HIV testing uptake amongst FSWs.

## Annex 1: Search Strategy

**Table:**

HIV (human Immunodeficiency virus) (AND combined with)	HIV testing/test/tested (AND combined with)	Sex work (AND combined with)
OR acquired immunodeficiency syndrome OR AIDS	OR voluntary counselling and testing OR VCT	OR people who sell sex
OR HIV OR human immunodeficiency virus	OR provider initiated testing and counselling OR PITC	OR sex industry/sex business
	OR provider initiated counselling and testing OR PICT	OR prostitution
	OR diagnostic/diagnosed	OR FSW OR female sex workers
	OR screening/screened	OR CSW OR commercial sex workers
	OR routine testing, Opt-In, Opt-Out	OR sex services
	OR positive result	OR escort services
	OR testing and counselling OR HTC	OR paid sex
		OR transactional sex

The final search strategy was defined as:

1. Search terms for HIV;
2. Search term for Sex workers;
3. Search term for HIV testing;
4. 1 AND 2 AND 3.

Date of search: 03/November/2017;

Searched fields: abstract, key words, subject headings, title;

Databases searched:

- Results: Ovid MEDLINE(R)-1,877studies
  - Embase-2516 studies
  - SCOPUS-2591 studies

1. HIV.ab,kw,sh,ti.
2. AIDS.ab,kw,sh,ti.
3. Human Immunodeficiency virus.ab,kw,sh,ti.
4. Acquired Immunodeficiency syndrome.ab,kw,sh,ti.
5. 1 or 2 or 3 or 4
6. “test* “.ab,kw,sh,ti.
7. “counsel* “.ab,kw,sh,ti.
8. HTC.ab,kw,sh,ti.
9. VCT.ab,kw,sh,ti.
10. (Voluntary counsel* and test*).ab,kw,sh,ti.
11. (Provider Initiated test* and counsel*).ab,kw,sh,ti.
12. PITC.ab,kw,sh,ti.
13. PICT.ab,kw,sh,ti.
14. (Provider Initiated counsel* and test*).ab,kw,sh,ti.
15. “diagnos* “.ab,kw,sh,ti.
16. “screen* “.ab,kw,sh,ti.
17. “Routine test* “.ab,kw,sh,ti.
18. Opt-In.ab,kw,sh,ti.
19. OPt-Out.ab,kw,sh,ti.
20. Positive results.ab,kw,sh,ti.
21. 6 or 7 or 8 or 9 or 10 or 11 or 12 or 13 or 14 or 15 or 16 or 17 or 18 or 19 or 20
22. “Sex* work* “.ab,kw,sh,ti.
23. “people who sell sex* “.ab,kw,sh,ti.
24. “sex* industry “.ab,kw,sh,ti.
25. “sex* business “.ab,kw,sh,ti.
26. “sex* service* “.ab,kw,sh,ti.
27. “prostitut* “.ab,kw,sh,ti.
28. “female sex* work* “.ab,kw,sh,ti.
29. “commercial sex* work* “.ab,kw,sh,ti.
30. “Escort* service* “.ab,kw,sh,ti.
31. “paid sex* “.ab,kw,sh,ti.
32. “transactional sex* “.ab,kw,sh,ti.
33. “FSW* “.ab,kw,sh,ti.
34. “CSW* “.ab,kw,sh,ti.
35. 22 or 23 or 24 or 25 or 26 or 27 or 28 or 29 or 30 or 31 or 32 or 33 or 34
36. 5 and 21 and 35
37. (“2000” or “2001” or “2002” or “2003” or “2004” or “2005” or “2006” or “2007” or “2008” or “2009” or “2010” or “2011” or “2012” or “2013” or “2014” or “2015” or “2016” or “2017”).yr.
38. 36 and 37

## Annex 2: Data Extraction Form

**Table:**

Title
Date published
Date of research
Authors
Study design
Research objectives/research questions
Country
Study population
Main methods of research
Main results
Main conclusions
Comments*(if needed)

## Annex 3: Quality Assessment

The quality of the qualitative papers (Table 1) was assessed using the guide for critically appraising qualitative research [15].The checklist consisted of 18 items assessing findings, design, sample, data collection, analysis, reporting, reflexivity and neutrality, ethics and auditability. As in previous research [66] we used a midpoint score of 9 as a cut off between low- and high-quality studies.

We assessed the quality of the quantitative and mixed-methods papers using the modified *Downs and Black* checklist [16]. The 26 questions of the checklist represented items of reporting, external and internal validity, and power. As the majority of studies did not report power calculations of the sample size and none was single or double blinded, we excluded the power (#27) and the blinding (#13, 14) questions. Thus, the modified checklist consisted of 24 questions with a maximum score of 25 points). A midpoint score of 12.5 was considered to distinguish the high-quality studies (Table 2).

**Table 2:** Quality assessment of the 36 studies

Study reference	Study design	Summary score for quality assessment
Qualitative methods (Spencer et al. [15])		
Ameyan et al. [47]	QL	61% (11/18)
Beattie et al. [48]	QL	72% (13/18)
Burke et al. [54]	QL	61% (11/18)
Chanda et al. [49]	QL	67% (12/18)
Dugas et al. [29]	QL	50% (9/18)
King et al. [46]	QL	50% (9/18)
Ngo et al. [50]	QL	72% (13/18)
Scorgie et al. [52]	QL	67% (12/18)
Wang et al. [41]	QL	67% (12/18)
Wanyenze et al. [51]	QL	72% (13/18)
Quantitative methods (modified Downs and Black [16])		
Batona et al. [28]	QN	56% (14/25)
Bengtson et al. [27]	QN	52% (13/25)
Chiao et al. [45]	QN	72% (18/25)
Dandona et al. [18]	QN	40% (10/25)
Deering et al. [25]	QN	60% (15/25)
Grayman et al. [30]	QN	48% (12/25)
Hong et al. [31]	QN	52% (13/25)
Johnston et al. [40]	QN	64% (16/25)
King et al. [46]	QN	52% (13/25)
Nhurod et al. [43]	QN	56% (14/25)
Parriault et al. [17]	QN	36% (9/25)
Shokoohi et al. [35]	QN	56% (14/25)
Shokoohi et al. [35]	QN	60% (15/25)
Todd et al. [32]	QN	52% (13/25)
Tran et al. [33]	QN	52% (13/25)
Wang et al. [19]	QN	48% (12/25)
Wang et al. [20]	QN	48% (12/25)
Wilson et al. [21]	QN, mathematical modeling	n/a
Xun et al. [42]	QN	56% (14/25)
Xu et al. [23]	QN	64% (16/25)
Mixed methods (modified Downs and Black [16])		
Aho et al. [26]	QN & QL	68% (17/25)
King et al. [34]	QN & QL	48 (12/25)
Abstracts		
Deering et al. [37]	QN	n/a
Park et al. [38]	QN	n/a
Sayarifard et al. [36]	QN	n/a
Simonovikj et al. [53]	Case report	n/a

*QL* qualitative, *QN* quantitative

## Annex 4: PRISMA 2009 checklist

**Table:**

Section/topic	#	Checklist item	Reported (Yes/No)
Title			
Title	1	Identify the report as a systematic review, meta-analysis, or both.	Yes
Abstract			
Structured summary	2	Provide a structured summary including, as applicable: background; objectives; data sources; study eligibility criteria, participants, and interventions; study appraisal and synthesis methods; results; limitations; conclusions and implications of key findings; systematic review registration number.	Yes
Introduction			
Rationale	3	Describe the rationale for the review in the context of what is already known.	Yes
Objectives	4	Provide an explicit statement of questions being addressed with reference to participants, interventions, comparisons, outcomes, and study design (PICOS).	Not applicable
Methods			
Protocol and registration	5	Indicate if a review protocol exists, if and where it can be accessed (e.g., Web address), and, if available, provide registration information including registration number.	Not available
Eligibility criteria	6	Specify study characteristics (e.g., PICOS, length of follow-up) and report characteristics (e.g., years considered, language, publication status) used as criteria for eligibility, giving rationale.	Yes
Information sources	7	Describe all information sources (e.g., databases with dates of coverage, contact with study authors to identify additional studies) in the search and date last searched.	Yes
Search	8	Present full electronic search strategy for at least one database, including any limits used, such that it could be repeated.	Yes (“Annex 1: Search strategy”)
Study selection	9	State the process for selecting studies (i.e., screening, eligibility, included in systematic review, and, if applicable, included in the meta-analysis).	Yes (Fig. 1)
Data collection process	10	Describe method of data extraction from reports (e.g., piloted forms, independently, in duplicate) and any processes for obtaining and confirming data from investigators.	Yes (“Annex 2: Data extraction form”)
Data items	11	List and define all variables for which data were sought (e.g., PICOS, funding sources) and any assumptions and simplifications made.	Yes
Risk of bias in individual studies	12	Describe methods used for assessing risk of bias of individual studies (including specification of whether this was done at the study or outcome level), and how this information is to be used in any data synthesis.	Not available
Summary measures	13	State the principal summary measures (e.g., risk ratio, difference in means).	Yes
Synthesis of results	14	Describe the methods of handling data and combining results of studies, if done, including measures of consistency (e.g., I^2^) for each meta-analysis.	Not applicable
Risk of bias across studies	15	Specify any assessment of risk of bias that may affect the cumulative evidence (e.g., publication bias, selective reporting within studies).	Yes
Additional analyses	16	Describe methods of additional analyses (e.g., sensitivity or subgroup analyses, meta-regression), if done, indicating which were pre-specified.	Not available
Results			
Study selection	17	Give numbers of studies screened, assessed for eligibility, and included in the review, with reasons for exclusions at each stage, ideally with a flow diagram.	Yes (Fig. 1)
Study characteristics	18	For each study, present characteristics for which data were extracted (e.g., study size, PICOS, follow-up period) and provide the citations.	Yes (Table 1)
Risk of bias within studies	19	Present data on risk of bias of each study and, if available, any outcome level assessment (see item 12).	Not available
Results of individual studies	20	For all outcomes considered (benefits or harms), present, for each study: (a) simple summary data for each intervention group (b) effect estimates and confidence intervals, ideally with a forest plot.	Not applicable
Synthesis of results	21	Present results of each meta-analysis done, including confidence intervals and measures of consistency.	Not applicable
Risk of bias across studies	22	Present results of any assessment of risk of bias across studies (see Item 15).	Yes
Additional analysis	23	Give results of additional analyses, if done (e.g., sensitivity or subgroup analyses, meta-regression [see Item 16]).	Not available
Discussion			
Summary of evidence	24	Summarize the main findings including the strength of evidence for each main outcome; consider their relevance to key groups (e.g., healthcare providers, users, and policy makers).	Yes (Table 1)
Limitations	25	Discuss limitations at study and outcome level (e.g., risk of bias), and at review-level (e.g., incomplete retrieval of identified research, reporting bias).	Yes
Conclusions	26	Provide a general interpretation of the results in the context of other evidence, and implications for future research.	Yes
Funding			
Funding	27	Describe sources of funding for the systematic review and other support (e.g., supply of data); role of funders for the systematic review.	Not available

Moher et al. [71]. For more information, visit: www.prisma-statement.org.
